# The use of annual killifish in the biocontrol of the aquatic stages of mosquitoes in temporary bodies of fresh water; a potential new tool in vector control

**DOI:** 10.1186/1756-3305-3-46

**Published:** 2010-05-21

**Authors:** Jonathan R Matias, Araceli Q Adrias

**Affiliations:** 1 Biomedical Sciences Laboratory, Poseidon Science Foundation, 122 East 42nd St., Suite 1700, New York, 10168, NY, USA; 2 Biocontrol R&D Laboratory, Nova Pacific, Miagao, Iloilo 5023 Philippines

## Abstract

**Background:**

Mosquitoes that breed in temporary pools in remote areas that dry up seasonally are especially difficult to control through chemical or biological means. The annual killifish has been suggested as a means of eradicating the aquatic stages of mosquitoes in transient pools because they can maintain permanent populations in such habitats by undergoing suspended animation or diapause during the embryonic stages to survive periodic drought. However, very little is known about the predatory activity of annual killifish and their usefulness in mosquito control.

**Results:**

The annual killifish, *Nothobranchius guentheri*, native to Tanzania, was used in this investigation. Food preference was tested under laboratory conditions by feeding juvenile killifish with 2^nd ^instar mosquito larvae of *Culex quinquefasciatus *in the presence of alternative food sources, such as rotifers and chironomid larvae. Semi-field tests were conducted by introduction of hibernating killifish embryos and juvenile fish to artificial ponds in an outdoor open environment that allowed natural oviposition of *Cx. quinquefasciatus*. Food preference studies show that *N. guentheri *preferred to prey on mosquito larvae than either chironomid or rotifers. When hibernating killifish embryos were added to ponds simultaneously with the addition of freshwater, the embryos hatched and fed on mosquito larval population resulting in complete elimination of the immature stages. The introduction of juvenile fish to ponds with high density of mosquito larvae resulted in total eradication of the mosquito population due to predation by fish. Complete biocontrol of the mosquito larval population was achieved in the presence of 3 fish per m^2 ^of pond surface area.

**Conclusions:**

The annual killifish provides yet another tool that may be employed in the eradication diseases carried by mosquitoes through vector control, particularly in temporary bodies of freshwater. The fish can be conveniently transported in the absence of water in the form of hibernating embryos. Once introduced either as embryos or juveniles in ponds, the annual killifish can effectively reduce the larval population because of its aggressive predatory activity.

## Background

Vector control using pesticides remains an important component of all mosquito control program worldwide. However, the persistent use of pesticides caused the development of chemically resistant substrains of mosquitoes. This chemical resistance in mosquitoes is increasing with new reports of resistance emerging in various areas [1-3]. For this reason and because of the ecosystem damage caused by such chemicals, there has been renewed interest in biological control techniques to complement mosquito control programs. The use of larvivorous fish is the oldest biocontrol method that is still employed today. The mosquito fish (*Gambusia affinis*), native to northeastern United States, was eventually selected for worldwide mosquito control because of its high larvivorous capacity, high fecundity and adaptability to new environments. However, the success of *Gambusia *in mosquito control was overshadowed by its intrinsic aggressive nature that drove many other native species of aquatic organisms to the brink of extinction [4]. More recent efforts had been focused on developing indigenous fish and other less aggressive aquarium fish as alternative to *Gambusia *in providing the same mosquito larval control in permanents streams and pools [5-7].

Mosquito larval control in breeding sites comprising pools that dry up seasonally limits the use of conventional fish. Transporting fish to remote areas is both logistically challenging and expensive. For these reasons, attention had been focused on a unique group of freshwater fish, collectively known as annual killifish, native to Africa and South America that can maintain populations in seasonal pools. These killifish can survive prolonged drought, sometimes lasting as much as five years, by undergoing suspended animation or diapause at three stages of their embryonic development [8,9]. At the end of the wet season, the adults die and the population survives in the form of diapausing embryos encased in the dry substrate [10]. These diapause embryos are more resistant to environmental extremes, such as high temperature, anoxia and physical/chemical damage, compared to non-diapause stages [10-14]. At the start of the first rains, the embryos hatch to re-populate the seasonal pools [15]. This life cycle mimics their main food source, which are mosquitoes and other insects that also begin their life cycle in the same seasonal pools. This convergent evolution provides a compelling reason for the interest in the potential of annual killifish for vector control in seasonal habitats.

The first record of annual killifish being used for this purpose was during World War II when Vanderplank initiated the introduction of *Nothobranchius taeniopygus *to combat malaria in seasonal swamps in Tanganyika and Kenya [16]. In 1952, the noted ichthyologist, George Myers, observed that the annual killifish may have potential in biological control since mosquito bites were diminished in areas normally populated by these fish [17]. Years later, others have made similar recommendations [18,19]. Some preliminary attempts made by the World Health Organization to use the annual killifish for mosquito control provided some information about the conditions necessary for proper introduction of annual killifish [18,20]. However, the limited financial support for a long term biocontrol research and inadequate knowledge about the environmental factors that control diapause had presented operational difficulties.

Much more is now known about the life cycle of *Nothobranchius guentheri*, an annual killifish indigenous to Tanzania. A hypothesis regarding its survival strategy in nature has already been proposed [21]. What is lacking before any serious attempts at field introductions is basic information regarding the larvivorous nature of these killifish. As part of an ongoing program to develop the use of annual killifish for mosquito control, the laboratory and semi-field studies in this report are designed to explore the larvivorous potential of *N. guentheri *and ways these fish might be employed in mosquito control.

## Methods

### Fish rearing

The breeding population of the annual killifish, *Nothobranchius guentheri*, was established at the Poseidon Sciences field laboratory in the Philippines where all the experiments described in this paper was conducted. This population was derived from the original population maintained in the United States since 1975 and interbred recently with *N. guentheri *breeding pairs supplied by Mr. Fred Behrman of Athens Aquatics (New York, USA) and Mr. Chris Butcher (Florida, USA). The killifish were maintained in 10 gallon aquaria according to previously described methods [22]. The spawning occurred in the tanks with substrate of fine sand. The fertilized eggs were collected and incubated in petri dishes at 25°C until they reach the pre-hatching state, where the embryo can remain quiescent for at most 3 months. Thereafter, they were transferred into moist sphagnum horticultural moss (Mosser Lee Co., Millston, Wisconsin, USA) at densities of 50 embryos per 25 g of moist peat moss in a transparent polyethylene plastic bag. After 30 days under incubation temperature of 25 ± 2°C, the embryos were competent to hatch and remained in the pre-hatching stage until placed in water for the hatching process to commence. Upon hatching, the fry were kept in glass aquaria and fed newly hatched *Artemia *for the first 2 weeks. Thereafter, the fry were fed live fruit fly larvae (*Drosophila melagonaster*) until they reach the juvenile size of 1.5 cm in length.

### Environmental conditions

Laboratory and semi-field studies were conducted in the island of Panay in the Philippines ( 10°40'25.17N, 121°57'39.47E) during the rainy season from May 2009 and ending in October, 2009. The water temperature ranged from 27° to 30°C, with pH in the range from 7.0 to 8.0. The dissolved oxygen level was 4 ppm. The artificial ponds were constructed near banana trees, with the overhanging leaves providing partial shade. The nearby trees also provided shade and leaves were permitted to fall naturally onto the experimental ponds. The only species of mosquitoes occurring naturally at this location was *Culex quinquefasciatus*. These mosquitoes were allowed to naturally oviposit in the experimental ponds. Other aquatic species present in the ponds in significant numbers were chironomids or midge larvae (*Chironomus plumosus*) and rotifers (*Brachionus sp.*).

### Laboratory studies

#### Feeding with rotifers and mosquito larvae

To study the food preference of *N. guentheri*, the prey organisms and the killifish were kept in glass tanks (11 cm depth × 29 cm length × 23 cm width) containing 7 liters of pond water. Freshwater rotifers were collected from an existing pond by passing the water through a fine mesh net. The concentrated freshwater rotifers were placed in the glass tanks filled with pond water. The rotifer count was determined by taking a 1 ml sample of the water and counting the number of rotifers under the microscope. 2^nd ^instar larvae of *Cx. quinquefasciatus *were collected from ponds and also introduced in the glass tank either separately or in combination with rotifers in the presence of 1 male fish, which was starved for 24 hours prior to the experiment. The rotifer and mosquito larval consumption was obtained at 30 min and 120 min after killifish introduction to the tank.

#### Feeding with chironomid and mosquito larvae

Chironomid larvae and 2^nd ^instar larvae of *Cx. quinquefasciatus *were manually collected from the artificial pond and introduced into the test aquaria with killifish as described above. The number of larvae remaining in the tank after a given time period was determined.

All laboratory tests were conducted during the light phase of the artificial photoperiod (14 h light phase: 10 hour dark phase) at water temperature of 27 ± 1.5°C. All freshwater used in this study was obtained from an artificial well.

### Semi-field studies

#### Measurements

All of the mosquito larvae and pupae were harvested from each test pond by using fine mesh dip nets. Given the small size of the test ponds, it was possible to harvest all mosquito larvae to obtain more reliable data on larval abundance. *Cx. quinquefasciatus*, the only mosquito species found in the pond, was characterized according to the stages (instars and pupae). The larvae and pupae were placed in shallow pans for counting and returned back to the pond within 3 hours. For simplicity, the total mosquito count represents all larvae and pupae combined. The chironomids were counted by determining the total number of intact cocoons found underneath leaves in the bottom substrate. The rate of growth of the annual killifish in the ponds was determined by measuring the standard length as the distance from the tip of the snout to the end of the caudal peduncle.

#### Effect of introduction of annual killifish fish embryos

To determine if newly introduced embryos of *N. guentheri *can hatch, survive and feed on mosquito larvae, 1 m × 1 m × 0.3 m plastic lined ponds were constructed using 0.75 mm polyethylene plastic sheeting. These artificial ponds were filled with freshwater initially to a height of 5 cm. The killifish embryos in peat moss were introduced to the pond on the same day. Each group is composed of 5 ponds. No other food was provided to the fries and the pond water was allowed to be augmented by the daily rains to a final height of 0.3 m. The bottom substrate was composed of sandy-muddy soil at a thickness of 1 cm. *Cx. quinquefasciatus *was allowed to naturally oviposit eggs simultaneously with the introduction of the fish embryos.

#### Effect of the introduction of juvenile killifish

Artificial ponds (1 m × 2 m) were constructed and filled with water to height of 0.3 m. *Cx. quinquefasciatus *was allowed to naturally oviposit in the ponds. After one week, the ponds showed substantial presence of mosquito larvae at which time a total of 5 juvenile killifish at 25 days of age (3 females and 2 males) measuring 1.5 cm in length were placed in each pond. The total number of mosquito larvae and pupae was determined at weekly intervals. The controls represented ponds that did not receive any killifish. Each test group consisted of 3 ponds. After 28 days, the killifish in the experimental ponds were removed to determine the time for mosquito population to recover in the absence of fish. Also at day 28, five killifish (3 females and 2 males) were added to the control ponds to determine the effect of the killifish in ponds with an already high mosquito density. No mortalities were observed during the transfer of the killifish and during length measurements.

To determine the fish density required to eradicate mosquito larval population in the ponds, juvenile male annual killifish were added to 1 m^2 ^ponds at densities from 0 to 5 fish per pond. Each test group was composed of 5 ponds. *Cx. quinquefasciatus *was allowed to naturally oviposit eggs for 1 week to provide a natural population of mosquito larvae. The total number of larvae present in the ponds was determined 4 days after fish introduction.

### Statistics

The statistical significance between control and experimental groups was evaluated using Student's t test.

## Results

**Figure 1:**
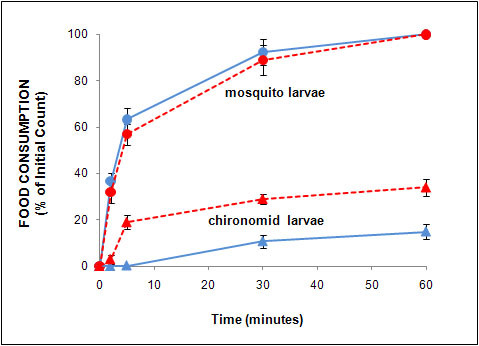
**The feeding preference by the annual killifish, *N. guentheri*, on mosquito and chironomid larvae under laboratory conditions**. Blue line represents food consumption when both types of larvae were given in combination. Red line represents food consumption when either of the two types of larvae was given as prey. Mean ± SEM of 5 tests per group.

Table 1 shows the preference of the annual killifish, *N. guentheri*, when presented with mosquito larvae and rotifers. There was no difference between males and females as far as food preference was concerned when corrected for body size (data not shown). The data for both sexes were therefore combined. When mosquito larvae and rotifers were given simultaneously as food source, the killifish preferentially consumed the mosquito larvae. After 120 min, the average consumption of mosquito larvae was 61.3% while only 24.1% of the rotifers were eaten. However, when presented with rotifers alone, the killifish doubled their consumption of rotifers. The difference in the food consumption between the rotifers alone and rotifers provided simultaneously with mosquito larvae were statistically significant (P < 0.0001). When presented with mosquito larvae alone, the killifish consumed the same amount of larvae as those killifish given both rotifers and larvae in combination. A similar pattern was observed when killifish was presented with mosquito larvae alone or in combination with chironomid larvae (Fig. 1). The annual killifish consumed 100% of the mosquito larvae within 60 min while eating only 12% of the chironomid larvae. But, when only chironomid larvae were given, the annual killifish doubled its consumption of the chironomids (P < 0.001).

**Table 1:** Food preference of male *N. guentheri *in the presence of rotifers and mosquito larvae as prey.

Test Groups	Mosquito larvae consumed (% of initial count)		Rotifer consumed (% of initial count)	
	
	30 min	120 min	30 min	120 min
Mosquito larvae + rotifers	33.3 ± 3.3	61.3 ± 4.4	7.3 ± 3.3	24.1 ± 5.4

Mosquito larvae alone	32.0 ± 3.2	69.6 ± 4.5	---------	----------

Rotifers alone	----------	-----------	30.3 ± 3.8	53.3 ± 3.9

The individual killifish was provided with 50 mosquito larvae in the presence of rotifers at initial density of 7.5 rotifers per ml. The data are presented as % consumed ± SEM after 30 min and 120 min. N = 6 fish for each test group.

**Figure 2:**
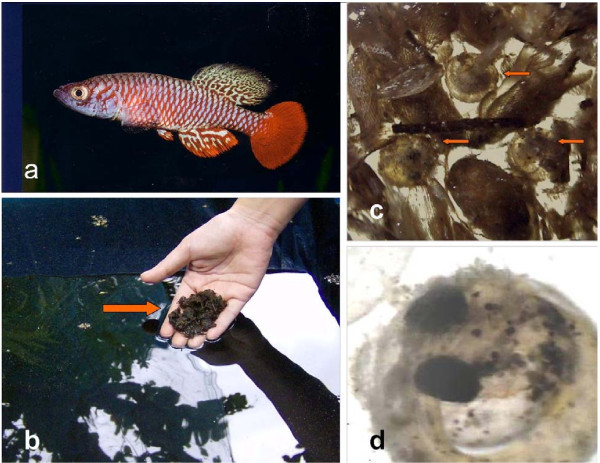
**Photographs showing (a) an adult male *N. guentheri*, (b) moist peat moss containing *N. guentheri *embryos being added to the pond water, (c) pre-hatching *N. guentheri *embryos within the peat moss, and (d) close-up of the pre-hatching embryo**.

**Figure 3:**
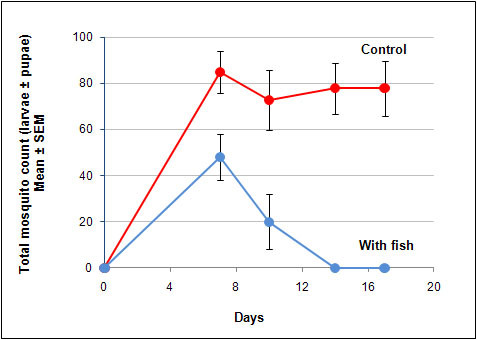
**The effect of the addition of pre-hatching embryos of the annual killifish, *N. guentheri*, on the mosquito larval population in ponds**. The data are presented as the total number of larvae and pupae present in the ponds at different periods of inspection. Total of 5 ponds per group.

The viability of introducing pre-hatching embryos in peat as a means of biocontrol was undertaken under semi-field conditions. Photograph showing the peat moss being placed on the pond water surface is shown in Fig. 2. The peat remained in the surface for a few minutes and slowly sunk to the bottom over the course of the day. Fig. 2 also shows the fully developed embryos inside the peat moss. Typical hatching rate was in the range of 40 to 50% of the total number of pre-hatching embryos within the peat. The killifish fry can be seen swimming on the surface of the pond 24 hours after introduction of the peat. The fry normally do not feed for the first three days and relied on its yolk sac to supply its needs while continuing its development. By day 5, the killifish can be seen preying on the 2^nd ^instar mosquito larvae that were oviposited at the same time as the introduction of the killifish embryos. In Fig. 3 the first inspection of the experimental ponds showed reduced larval count on day 7 compared to the control pond (P < 0.01). While the larval count in the control ponds stabilized at about 80 larvae for the remainder of the study period, the ponds with introduced eggs declined in larval/pupal counts to 0 by day 14. It remained at 0 until the termination of the study on day 17.

**Figure 4:**
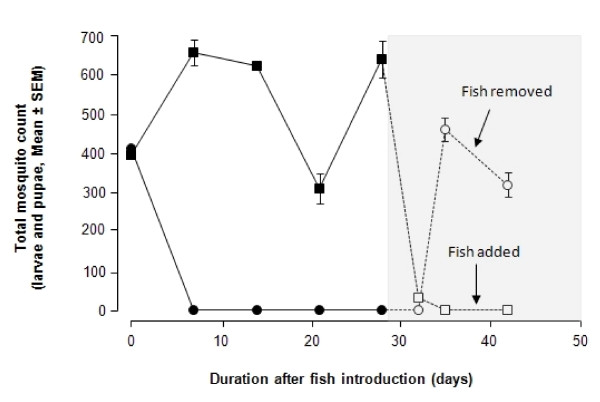
**The effect of the introduction of juvenile *N. guentheri *in ponds on mosquito larval count**. Each point represents data for triplicate ponds. Each experimental pond (closed solid circle) received a set of 2 males and 3 females. On day 28 all the killifish were removed from the experimental pond and the monitoring was continued in the absence of killifish (open circle). The control group (closed solid square) consisted of ponds without killifish. On day 28, 2 males and 3 females were added to the control ponds (open square). The shaded area represent the period when the conditions of the ponds were changed.

Fig. 4 shows the effect of introducing juvenile fish to a pond with pre-existing mosquito larval population. Controls consisted of ponds wherein no killifish was introduced. The total mosquito count in all 6 ponds at start of the experiment was 400, comprising 65% as 2^nd ^instar and 35% as 3^rd ^instar larvae. By week 2, further recruitment into the larval population continued with the appearance of pupae. The data show that all the mosquito larvae were decimated in the ponds one week after the introduction of the killifish. In contrast, the control ponds showed high mosquito counts for the following three weeks. At day 28, the killifish were removed from the experimental ponds. In the absence of fish these ponds developed a substantial population of mosquito larvae 8 days later. When killifish were introduced to control ponds that did not previously contain any killifish, the number of larvae precipitously dropped to 0 and remained at that level throughout the duration of the experiment.

**Figure 5:**
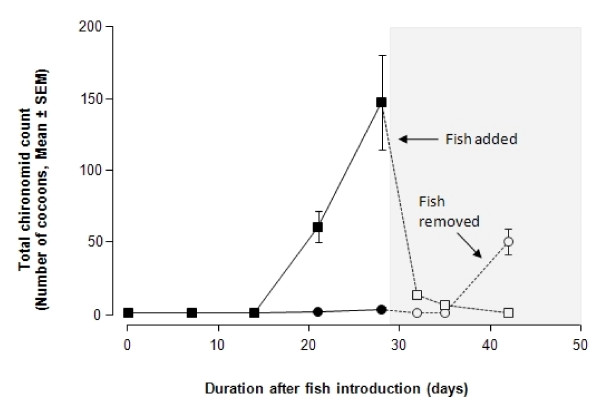
**The effect of the introduction of juvenile *N. guentheri *in ponds on chironomid larval count**. The experimental conditions are identical to that shown in Fig. 4. Each point represents data for triplicate ponds. Each experimental pond (solid black circle) received a set of 2 males and 3 females. On day 28 all the killifish were removed from the experimental pond and the monitoring was continued in the absence of killifish (open circle). The control group (closed solid square) consisted of ponds without killifish. On day 28, 2 males and 3 females were added to the control ponds (open square). The shaded area represent the period when the conditions of the ponds were changed.

The chironomid count was also assessed in the same ponds. There was a 14-day delay before chironomids can be observed in the control ponds. This was followed by an increase in the number of chironomid larvae thereafter (Fig. 5). In contrast, the ponds with killifish had no chironomids during the first 28 days of the study. When the killifish were removed from the experimental ponds, the presence of chironomids became evident at 2 weeks after removal of the killifish. Upon introduction of the killifish to the control pond at day 28, the chironomid count dropped from 140 chironomids to 10 and continued its downward trend to 0 by day 42.

**Figure 6:**
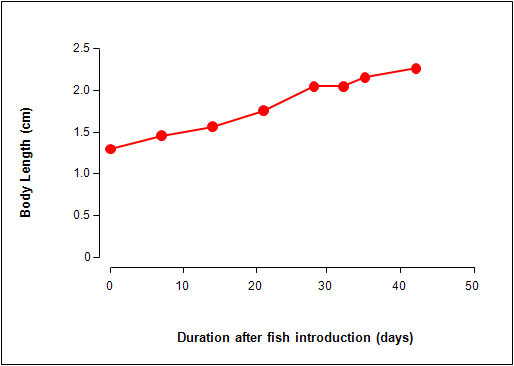
**Growth of *N. guentheri *during the 42-day study in the ponds**. The SEM for each data point was too small to be accurately plotted and therefore is not shown in this graph. Mean of 5 killifish.

**Figure 7:**
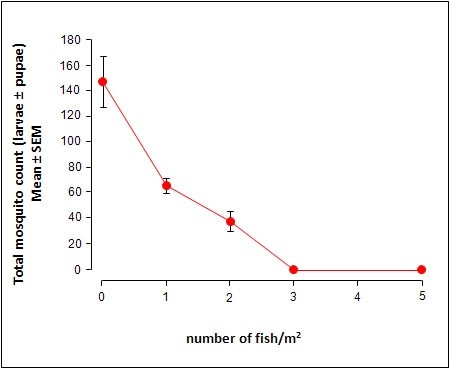
**The relationship between *N. guentheri *density and mosquito larval density in ponds**. Each data point represents 5 ponds per test group.

The data in Fig. 6 show the growth of killifish (3 females and 2 males) from the time of introduction to the end of the study in a single pond. The killifish grew well in the pond and the growth rate was much faster compared to those populations grown in aquarium tanks (data not shown). The coloration of the males was even more striking compared to aquarium grown killifish. Since the killifish were not provided with any additional food besides what was naturally available in the pond, the excellent growth rate suggests that there was sufficient food in the natural ponds for sustenance. The data in Fig. 7 show that the decline in mosquito density is dependent on killifish density, with total biocontrol possible within 4 days with just 3 juvenile killifish per m^2 ^of pond surface area.

## Discussion

The data described in this report validate the potential of using the annual killifish, *N. guentheri*, for mosquito control because of its preference for mosquito larvae as prey, the successful demonstration of introducing the fish in the ponds in the form of diapausing eggs and the eradication of mosquito larval population in the ponds. The successful use of fish in mosquito control has been described before. Gosh and Dash [23] has demonstrated success in controlling *An. culicifacies *in wells and in ponds in India using only fish as the method of control.

**Figure 8:**
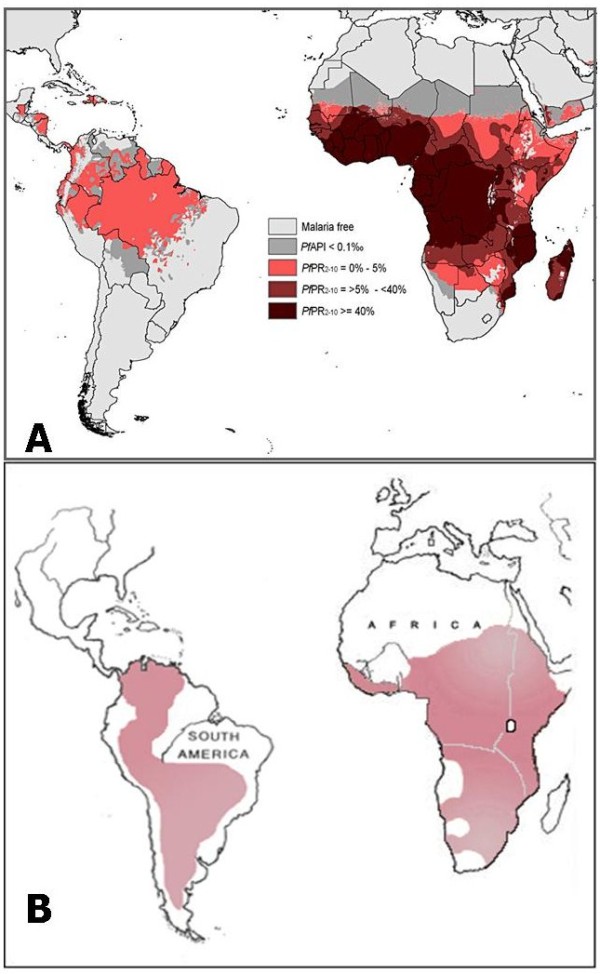
**Comparison of the geographic range of annual killifish species and malaria risk**. (A) Distribution map of malaria risk in Africa and South America (modified from Hay, *et al *[24]). (B) Distribution map of the annual killifish habitats in Africa and South America.

With the current emphasis on integrated vector management for malaria control the use of local species of annual killifish may represent an additional tool in the control of malaria vector species since the distribution of malaria [24] overlaps the geographic range of annual killifish species in South America and Africa (Fig. 8). It is therefore be possible to select a species native to a particular region that may be effectively employed for mosquito control. In this study we have only tested culicine mosquitoes and further work is needed to confirm the effectiveness of killifish against anopheline species. The genus *Nothobranchius *alone comprises at least 40 species distributed throughout Africa [25]. In natural depressions that collect water in Somalia as an example, the stomach contents of indigenous *Nothobranchius *species frequently contained larvae of mosquitoes (species not identified), lesser water boatmen (*Coryxa sp*.) and various small crustaceans [13]. Mosquito larva was the preferred prey based on analysis of gut contents. Our food preference studies indicate that this is the case in a laboratory setting. A similar pattern of food preference for culicine larva in the presence of other alternative invertebrate prey was found in three indigenous air-breathing fish in India considered for malaria control [26].

Food preference for mosquito larvae was evident when either chironomids or rotifers were simultaneously presented as prey. The size of the prey is not likely a major factor since the average size of the 2^nd ^instar mosquito larvae at 0.6 mm was not markedly different from the mean length for chironomid at larvae at 0.7 mm. Rotifers were much smaller prey measuring approximately 0.2 mm. The annual killifish would eat rotifers and chironomids as alternate food source if no other food was available, but overwhelmingly preferred mosquito larvae. Like most annual killifish, *N, guentheri *is a surface feeder and specially adapted to feed on aquatic invertebrates at or near the surface of the pond. The annual killifish will actively chase after the mosquito larvae upon sensing movements. Hence, the preference for mosquito larva that spends most of its time at the surface. In contrast, chironomids are bottom dwelling and typically found underneath decaying leaves. Rotifers are free swimming throughout the water column and likely preferred by the young killifish fry during the first week after hatching because of their small size.

The high predatory activity of *N. guentheri *on culicine larvae strongly suggests that it is a suitable candidate for mosquito control. The results in this study show that a minimum of 3 killifish is adequate to eradicate the larval population from 1 m^2 ^of pond surface area. Whether anopheline mosquitoes will be equally preyed upon like the culicine mosquitoes in this study or whether other annual killifish will have similar aggressive predatory activity remains to be established. Recently, Louca, et al [27] demonstrated that culicine mosquitoes avoid ovipositing in water containing fish. This may explain the total absence of new culicine mosquitoes in the present study after the introduced fish had decimated the larval and pupal stages. However, Anopheles *gambiae *do not share the same oviposition behavior and will continue to lay eggs in the presence of fish in the pond [27]. The present study is now being expanded to look into the predatory behavior of annual killifish in Tanzania where *An. gambiae *is endemic.

The onset of diapause sets the annual killifish apart from conventional fish and provides a novel means of providing biological control in transitory bodies of freshwater. The convenience of transporting and disseminating embryos for mosquito control purposes remain a major advantage particularly in remote areas where insecticide resistance is rampant and places where chemical use is not wanted by the local population. Even if re-introduction of new embryos becomes necessary during the next rainy season, this task can be easily accomplished by local mosquito control officers or even by the local population. The likelihood that introduced eggs will maintain sustainable populations in any new habitats is still open for exploration. The substrate in a typical *Nothobranchius *habitat is composed of black or gray mud, commonly referred to as "black cotton soil" or vertisols which consist of swelling clays, such as montmorillonite, with high water retaining capacity [18,28]. *Nothobranchius *is never observed to maintain populations in habitats with red clays or oxisols. This habitat requirement may limit the type of environments that may sustain *Nothobranchius *species.

There are, however, ecological issues of concern before annual killifish can be employed for this purpose. Introduction of *N. guentheri *to other areas, even within Tanzania, may pose some risk to contamination of other transient pools inhabited by other species of *Nothobranchius*. However, field studies have shown that in many instances *Nothobranchius *species can be found in natural settings already intermixed with other species of annual killifish [18,29]. While *N. guentheri *is used for this study merely as a demonstration of the potential of using annual killifish for mosquito control and should be limited for use in its natural geographic range, there are other species, such as *N. melanospilus*, that have a wider distribution in Tanzania that may be better adapted to the different conditions found in the country (B. Watters, personal communication).

Another issue of concern is whether introduction of this species in new habitats may pose risks to other indigenous aquatic organisms. Annual killifish survive only in temporary pools and require a period of drying to complete its life cycle. For this reason, annual killifish are never found in permanent bodies of water. Since they are typically the dominant inhabitant in a transient pool, annual killifish may not have evolved behavioral adaptations to compete against other fishes. In multi-species aquaria for example, annual killifish do not survive well. The aggressive nature of *N. guentheri *is only evident when males compete for the attention of females for reproduction and to establish dominance hierarchies [30]. While it is likely for introduced killifish to migrate to permanent waters due to flooding or transport of eggs by birds or other animals, it is quite unlikely that annual killifish can maintain permanent presence in such habitats. This question needs to be further elucidated with controlled studies to accurately determine the potential ecological impact of introducing annual killifish to new environments.

There are many practical advantages of the annual killifish in mosquito control. First, there is a wide range of indigenous species of annual fish found in South America and sub-Saharan Africa. It is therefore feasible to use indigenous annual killifish species for any given malarial region rather than introducing an exotic species to new habitats. Second, the use of diapausing embryos may allow in suitable areas to have recurring populations in transient bodies of freshwater so that re-introductions will not be necessary, thereby reducing cost of maintaining mosquito control. Vectors of malaria and dengue propagate in both permanent and in transient pools, such as tire tracks, water collecting stations, animal footprints and depressions created by mining activities. Transporting conventional live fish for biocontrol measures in such situations will be difficult and costly. On the other hand, transporting thousands of annual killifish embryos in peat moss or in a more convenient delivery system would make it easier and more economical. Third, the annual killifish are small and less likely to be used as food source. The small size also allows easy access to shallow areas where mosquito larvae tend to congregate. Fourth, the annual killifish is not aggressive against other fish, cannot survive in permanent bodies of freshwater and likely pose minimal ecological issues. And finally fifth, as shown in this report, the annual killifish is sufficiently larvivorous to exert substantial control of the immature stages of the mosquito population. All of these characteristics satisfy World Health Organization's recommendations on the selection of larvivorous fish for mosquito control [31].

## Conclusion

In summary, this study demonstrates that the annual killifish, *Nothobranchius guentheri*, aggressively preys on culicine larvae preferentially compared to other aquatic food sources under both laboratory and semi-field conditions. Total control of the immature stages of mosquitoes in freshwater can be achieved with the introduction of killifish. More important, introduction of hibernating or diapause embryos serves as a convenient means of disseminating killifish in transient pools. This study suggests that annual killifish may be useful in the future as part of the integrated program in vector control.

## Competing interests

The authors declare that they have no competing interests.

## Authors' contributions

JRM participated in the design of the study, writing the manuscript and performing the statistical analysis. AQA participated in its design, coordination and helped to draft the manuscript. All authors read and approved the final manuscript.
